# Vertigo in children: etiology and clinical characteristics of acute vestibular disorders

**DOI:** 10.3389/fneur.2026.1883728

**Published:** 2026-08-12

**Authors:** Patricia Alejandra Sommerfleck, Carlos Mario Martinez, Lucas Damian Costa, Sergio Carmona

**Affiliations:** 1 Hospital de Pediatria Prof Dr Juan P Garrahan, Buenos Aires, Argentina; 2 Hospital J. M. Cullen, Santa Fe, Argentina; 3 Universidad Nacional del Litoral Facultad de Ciencias Medicas, Santa Fe, Argentina; 4 Instituto de Neurociencias Buenos Aires, Buenos Aires, Argentina

**Keywords:** acute vestibular syndrome (AVS), childhood, dizziness, pediatric patients, vertigo

## Abstract

**Introduction:**

Acute vestibular syndrome (AVS) in children is an uncommon but clinically challenging presentation, often requiring rapid differentiation between peripheral and central causes. Evidence focusing specifically on AVS in the pediatric population using standardized vestibular classifications remains limited. The aim of this study was to describe the frequency, etiological distribution, and age-related patterns of acute vestibular syndrome in a large cohort of pediatric patients.

**Materials and method:**

We conducted a retrospective, descriptive, analytical and observational study including patients aged 0–18 years who presented with balance disorders at a single tertiary pediatric hospital between March 2015 and December 2022. Cases were classified according to the Bárány Society criteria into acute, episodic, and chronic vestibular syndromes. Clinical, demographic, and etiological data were analyzed, with a particular focus on AVS.

**Results:**

A total of 723 patients were included. AVS accounted for 18.3% of cases [95%CI: 15.5–21.2]. The most frequent etiologies were acute post-infectious cerebellitis (21.9%), vestibular neuritis (17.4%), and post-traumatic vertigo (15.9%). Overall, central causes were more frequent than peripheral etiologies (56.8% [95%CI: 48.3–65.0] vs. 43.2% [95%CI: 35.0–51.7]). A significant age-dependent shift was observed (χ^2^ = 11.5; *p* = 0.003): central causes predominated in children aged 0–5 years (73.7% [95%CI: 58.0–85.0]), while peripheral etiologies became more frequent in adolescents aged 12–18 years (63.4% [95%CI: 48.1–76.4]).

**Conclusion:**

AVS in pediatric patients is a heterogeneous entity with a clear age-dependent etiological pattern. Central causes predominated in younger children, whereas peripheral vestibular disorders became increasingly common with age. These findings highlight the importance of an age-tailored diagnostic approach in the evaluation of acute vertigo in children. Although the study population may be subject to selection bias, our findings may still assist clinicians in formulating the differential diagnosis when evaluating pediatric patients with AVS.

## 1 Introduction

Acute vestibular syndrome in children is an uncommon but clinically challenging condition, often requiring rapid differentiation between peripheral and central causes. Evidence focusing specifically on AVS in the pediatric population using standardized vestibular classifications remains limited.

Most vestibular disorders are episodic (1, 2), and it is the recurrence of these episodes that alerts parents and prompts specialist consultation.

Acute vestibular syndromes typically last several hours, and patients almost invariably present early to the emergency department. The symptoms are pronounced and abrupt, leading to timely medical evaluation. In contrast, brief episodes—such as those observed in benign paroxysmal vertigo of childhood (BPVC)—may initially be difficult for children to articulate due to their transient nature and may also be challenging for caregivers to recognize or consider clinically significant. This is particularly relevant because the first episode of conditions such as vestibular migraine or BPVC may nonetheless lead to evaluation in the emergency setting.

The vestibular system, especially the neural pathway, does not fully mature until early adolescence. In addition, pediatric patients have a significant capacity for adaptation and compensation due to their greater neural plasticity (3).

Consultations for balance diseases frequently require a multidisciplinary approach involving general pediatrics, neurology, otolaryngology, ophthalmology, cardiology, psychology, and often other specialties. The primary purpose is to identify the underlying etiology and establish an accurate diagnosis.

Dizziness in children may result from a broad spectrum of conditions. In this study, we describe the most common causes of balance disorders evaluated in a single tertiary pediatric hospital, at otolaryngology department, with particular emphasis on the acute causes of vertigo in pediatric patients. The aim of this study was to characterize the frequency, etiological distribution, and age-related patterns of acute vestibular syndrome in a large cohort of children and adolescents.

## 2 Materials and methods

### 2.1 Patients

Patients aged 0 to 18 years who presented with balance disorders to the Department of Otolaryngology at a single tertiary pediatric hospital between March 2015 and December 2022 were included in the study.

For the analysis of disorders according to age, we used the age-group classification criteria established by the World Health Organization and the American Academy of Pediatrics that were current at the time of data collection. Accordingly, patients were categorized into the following age groups: 0–5 years (preschool children), 6–11 years (school-aged children), and 12–18 years (adolescents).

### 2.2 Study design

A retrospective, descriptive, analytical, and observational study was conducted. Patients were consecutively enrolled at the time of their initial consultation.

The registry and medical records of all included patients were available, together with electronic access to consultations requested for diagnostic confirmation.

The study was conducted in accordance with the ethical principles outlined in the Declaration of Helsinki and was approved by the Medical Research Ethics Committee of Hospital de Pediatría Prof. Dr. Juan P. Garrahan.

### 2.3 Statistical analysis

Statistical analyses were performed using Python (Pandas, SciPy, and Seaborn libraries). Categorical variables were expressed as absolute frequencies and percentages. Confidence intervals for proportions were calculated using the Wilson method. Comparisons between age groups and etiological categories (peripheral vs. central) were conducted using the chi-square test. A two-sided significance level of *α* = 0.05 was adopted.

### 2.4 Vestibular studies

All patients underwent a detailed interview in the presence of a parent or caregiver, together with a comprehensive otoneurologic physical examination.

Vertigo is defined as the subjective sensation of rotational movement, either of oneself or of the surrounding environment. Children often described this sensation as the floor or room spinning, a feeling of self-rotation, or an experience similar to being on a merry-go-round or some other spinning object.

In contrast, dizziness refers to a more general sensation of imbalance or discomfort that is more challenging for patients to describe. Patients may report a sensation of a “heavy head,” walking on clouds, or feeling as though an external force were pushing them. It is a non-specific symptom and may be associated with systemic disorders.

Imbalance is a gait disorder that is often multisensory in origin, resulting from impairment of the visual, motor, sensory, or cerebellar systems. Symptoms improve or disappear when the patient is sitting or lying down.

Ataxia is a disorder of posture and control of voluntary movements, secondary to involvement of the cerebellum and cerebellar pathways. The most characteristic features of ataxic gait are a broad-based stance and motor incoordination, often accompanied by a sensation of imminent falling.

These symptoms were assessed through a standardized interview conducted by the same specialist in otoneurology for all patients.

The following clinical tests were performed: the Romberg test, Bárány index test, tandem gait assessment, Unterberger stepping test, evaluation of smooth pursuit and saccadic eye movements, assessment of dysmetria and diadochokinesia, and examination for spontaneous nystagmus.

Videonystagmography, including assessment of spontaneous nystagmus in the primary gaze position (0°) and positional testing, was conducted using Ecleris® VNG-PLUS equipment with a dual-camera system. The video head impulse test (vHIT)was performed using ICS Otometrics® equipment to assess vestibulo-ocular reflex gain in the horizontal semicircular canals. Both instrumented assessments were systematically performed in all patients older than 4 years. In younger children, a detailed clinical and family history, together with information provided by parents or caregivers, is essential. In some cases, videos recorded on mobile phones may provide valuable information and help clarify the diagnosis. Additional investigations, such as brain magnetic resonance imaging (MRI), electroencephalography (EEG), or other diagnostic tests, should be considered on a case-by-case basis. Careful follow-up of these patients is also essential.

For the evaluation of acute presentations, disorders were categorized according to their clinical presentation using the International Classification of Vestibular Disorders proposed by the Bárány Society (4).

Acute vestibular syndrome (AVS) was defined as acute-onset continuous vertigo lasting from hours to days, typically associated with nausea, vomiting, gait instability, nystagmus, and intolerance to head movements. Patients are usually symptomatic at the time of clinical evaluation, reflecting an acute and ongoing disorder of the vestibular system.

Episodic vestibular syndrome (EVS) was defined as recurrent, transient episodes of vertigo, dizziness, or imbalance, lasting from seconds to hours, often accompanied by symptoms or signs suggesting temporary vestibular dysfunction. Associated cochlear or central nervous system manifestations may be present. Episodes may be spontaneous or triggered by specific stimuli, and patients may present for evaluation even after a first symptomatic episode.

Chronic vestibular syndrome (CVS) was defined as a persistent vestibular disorder characterized by chronic vertigo, dizziness, or imbalance with gradual or continuous onset, lasting for weeks, months, or even years. The symptoms may remain stable or fluctuate, often overlapping, and are indicative of sustained vestibular system dysfunction.

Accurate differentiation between peripheral and central vestibular syndromes is essential, particularly in the evaluation of acute presentations. Baloh (5) clearly described the distinguishing features based on the characteristics of nystagmus, the vHIT, and ataxia.

Spontaneous nystagmus of peripheral origin is typically horizontal with a torsional (rotational) component and does not change direction with changes in gaze. In contrast, spontaneous nystagmus of central origin is often purely horizontal, vertical, or torsional and usually changes direction according to gaze position. The vHIT is abnormal when performed toward the affected side in peripheral vestibular lesions but is normal in central lesions.

Patients with an acute peripheral vestibular lesion are generally able to stand, although they tend to veer toward the affected side. However, patients with vertigo of central origin are often unable to stand without support. Associated neurological signs, including dysarthria, incoordination, numbness, or weakness suggest a central origin. Conversely, auditory symptoms, such as hearing loss, tinnitus, or ear pressure, support a peripheral cause.

These clinical principles are fundamental for the differential diagnosis of pediatric patients. Distinguishing central from peripheral signs is essential. In settings where complementary exams, such as the video head impulse test (vHIT) or brain magnetic resonance imaging (MRI), are unavailable or cannot be routinely performed, careful clinical assessment and longitudinal evaluation remain the mainstays of diagnosis.

The clinical criteria and complementary investigations used to establish the most relevant diagnoses in this study are outlined below:

Post-infectious acute cerebellitis: In children younger than 5 years, the clinical presentation is the cornerstone of the diagnosis. These patients typically present with the sudden onset of ataxia without vertigo, autonomic symptoms, or auditory symptoms, often accompanied by dysmetria and a broad-based gait. A history of a recent viral illness is an important diagnostic clue, and the generally benign clinical course with progressive symptom resolution further supports the diagnosis. Careful consideration of alternative causes of acute ataxia is essential when establishing the differential diagnosis. Brain MRI was performed only when clinically indicated (persistent symptoms, atypical presentation, focal neurological deficits, or diagnostic uncertainty), rather than routinely in all patients with suspected post-infectious cerebellitis. Close follow-ups are fundamental.

Vestibular neuritis: The diagnosis was based on a detailed clinical history, including a preceding viral illness within the previous 15–20 days, acute vertigo lasting several hours, associated autonomic symptoms, and the absence of auditory symptoms. Physical examination findings were consistent with acute unilateral vestibular hypofunction, which was confirmed by the video head impulse test (vHIT) demonstrating unilateral vestibular hypofunction. According to our database, all patients diagnosed with vestibular neuritis were 5 years of age or older, allowing vHIT to be successfully performed and the diagnosis objectively documented.

Acute labyrinthitis: As this was an Otorhinolaryngology (ENT) department, acute otitis media could be readily identified. In several cases, the presence of a retrotympanic effusion prompted tympanocentesis, allowing microbiological identification of the causative pathogen.

Post-traumatic vertigo: Patients presented with a recent history of mild traumatic brain injury, without any alternative explanation for their acute vestibular symptoms. All patients underwent a comprehensive vestibular evaluation, including bedside examination and instrumental vestibular testing when feasible, and all demonstrated normal vestibular function, with no evidence of peripheral vestibular injury. Cases in which head trauma resulted in benign paroxysmal positional vertigo (BPPV) were classified separately as BPPV rather than post-traumatic vertigo. Only one patient sustained a temporal bone fracture associated with sensorineural hearing loss, which was classified separately. Patients with post-traumatic vertigo were classified as having presumed central vestibular disorders only when no objective evidence of peripheral vestibular dysfunction was identified on bedside examination or complementary vestibular testing. We acknowledge that the absence of peripheral findings does not definitively establish a central origin, and this classification was adopted solely for analytical purposes.

## 3 Results

During the study period, approximately 60,000 visits were registered in the Department of Otolaryngology at a tertiary pediatric hospital. Of these, around 2,500 visits were related to balance disorders, representing approximately 4.1% of all otolaryngology consultations.

**Figure 1:**
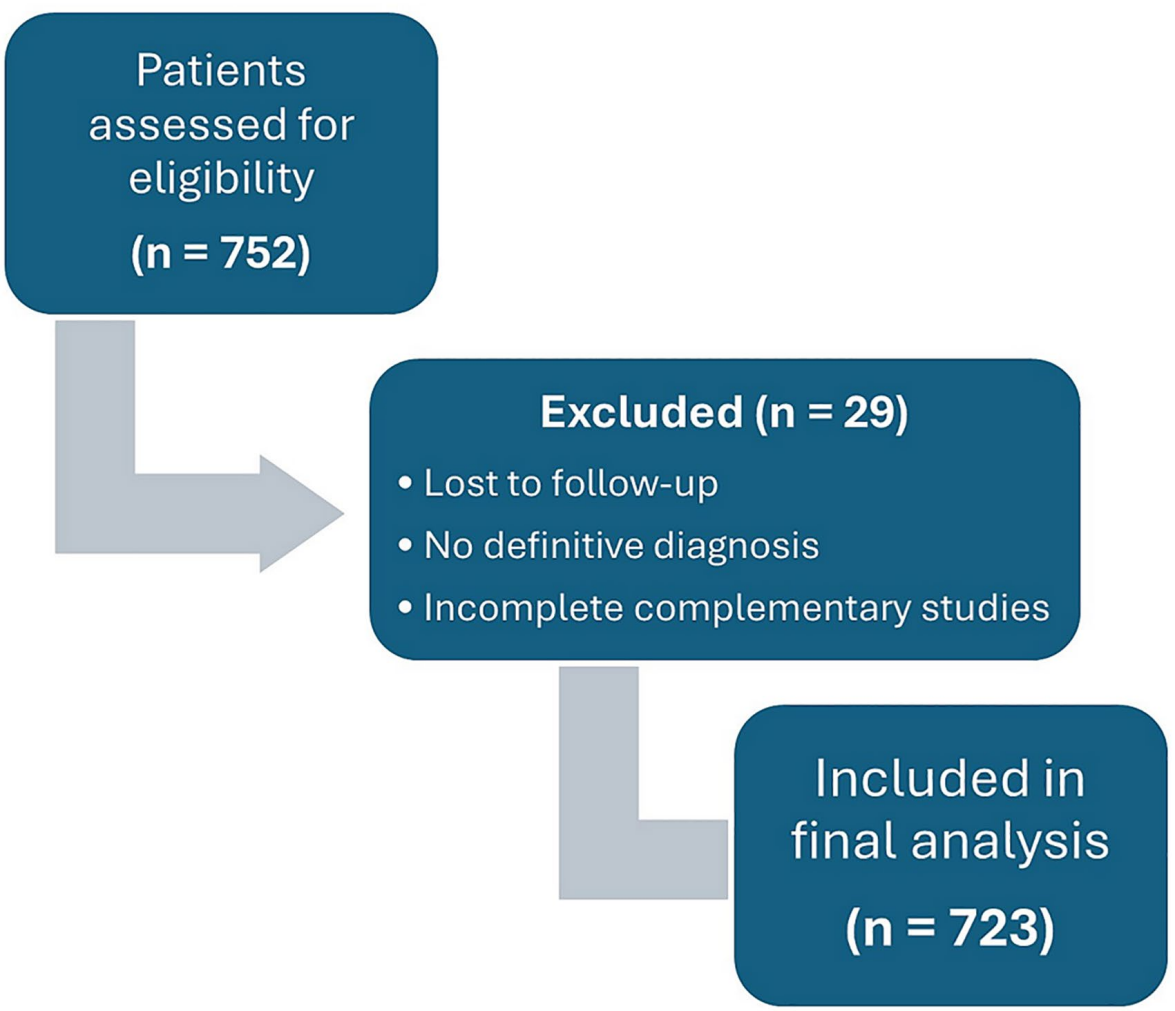
Flow diagram of patient selection and inclusion in the study. The figure illustrates the relationship between the total number of consultations for balance disorders and the final cohort included in the analysis.

A total of 752 patients were initially assessed for eligibility during the study period. After excluding 29 patients due to loss of follow-up, absence of a definitive diagnosis, or incomplete complementary studies, the final cohort included 723 patients for analysis (Figure 1). The main baseline characteristics of the study population are summarized in Table 1.

**Table 1:** Baseline characteristics of the study population.

Variable	Total (*n* = 723)
Sex	
Female	369 (51.1%)
Male	354 (48.9%)
Age groups	
0–5 years	172 (23.8%)
6–11 years	321 (44.4%)
12–18 years	230 (31.8%)
Most frequent individual diagnoses	
Benign paroxysmal vertigo of childhood	96 (13.3%)
Primary headache	84 (11.7%)
Vestibular migraine	73 (10.1%)
Functional disorders	71 (9.9%)
Acute post-infectious cerebellitis	29 (4.0%)
Vestibular neuritis	27 (3.8%)
Post-traumatic vertigo	22 (3.1%)

The cohort shows a balanced sex distribution and a predominance of school-aged children.

The reported figure of 2,500 reflects the total number of consultations for balance disorders, whereas only 752–723 individual patients were included in the analysis. This discrepancy is explained by the longitudinal nature of care the difference between consultations and individual patients, as many patients attended multiple follow-up visits throughout the study period.

The cohort showed a balanced sex distribution, with 369 females (51.1%) and 354 males (48.9%). For analytical purposes, patients were categorized into three clinically relevant age groups: 0–5 years (*n* = 172; 23.8%), 6–11 years (*n* = 321; 44.4%), and 12–18 years (*n* = 230; 31.8%).

**Figure 2:**
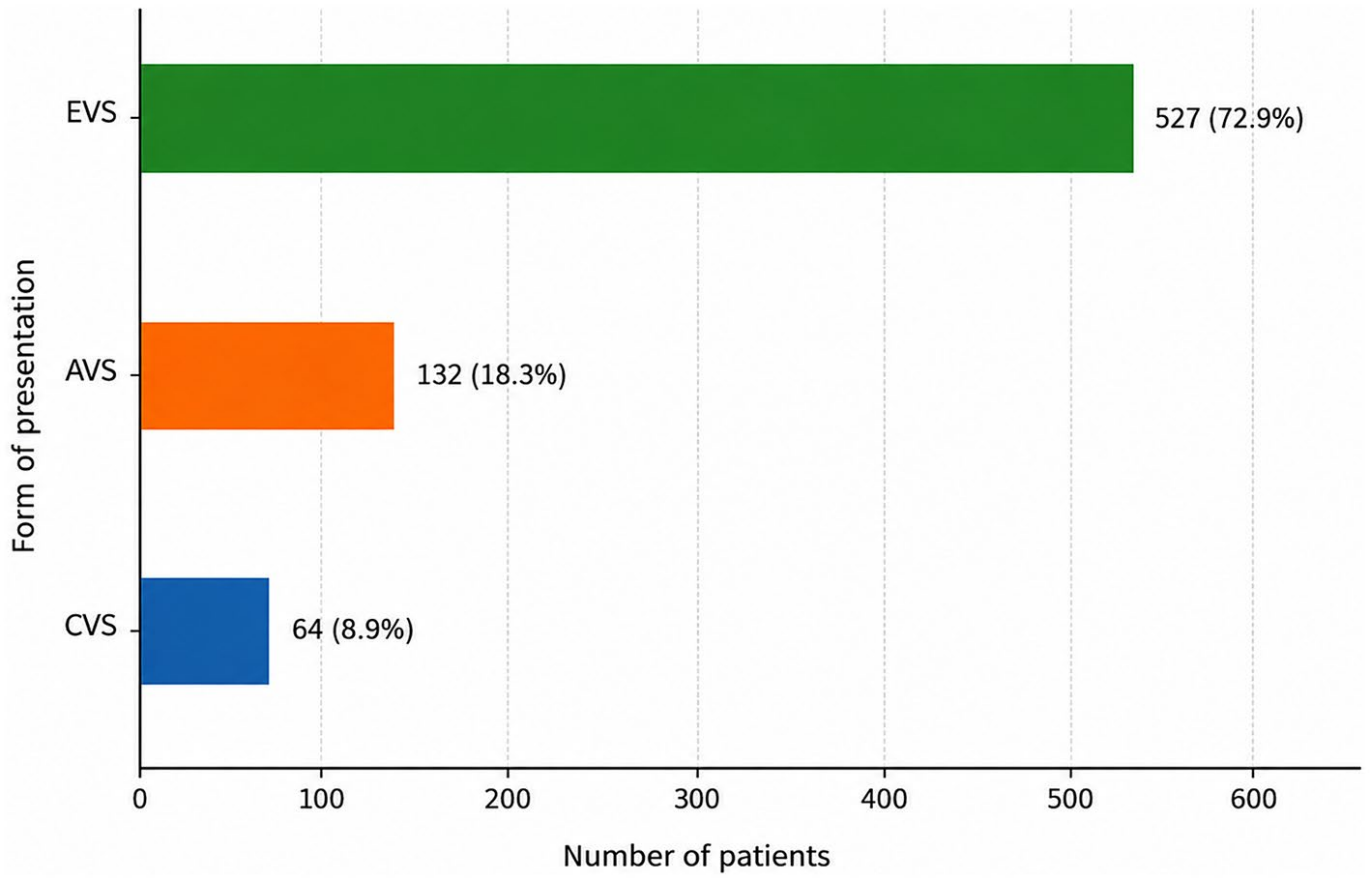
Distribution of vestibular syndromes according to clinical presentation. Although episodic syndromes predominate, AVS represents a clinically relevant subset.

The distribution of vestibular syndromes according to clinical presentation is shown in Figure 2. AVS was identified in 132 patients (18.3% [95%CI: 15.5–21.2]), EVS in 527 patients (72.9% [95%CI: 69.5–76.0]), and CVS in 64 patients (8.9% [95%CI: 7.0–11.1]).

### 3.1 Acute vestibular syndrome: overall frequency and etiological profile

AVS was identified in 132 of the 723 included patients (18.3%). Within this subgroup, 64 patients were female (48.5%) and 68 were male (51.5%). When analyzed by age, AVS cases were distributed as follows: 38 patients were aged 0–5 years (28.8%), 53 were aged 6–11 years (40.1%), and 41 were aged 12–18 years (31.1%). Thus, although episodic vestibular syndromes predominated in the overall cohort, AVS accounted for a relevant proportion of consultations across all pediatric age groups.

Among patients with AVS, the most frequent diagnoses were acute post-infectious cerebellitis (29/132; 21.9%), vestibular neuritis (23/132; 17.4%), post-traumatic vertigo (21/132; 15.9%), sudden hearing loss (12/132; 9.1%), labyrinthitis (11/132; 8.3%), and post-vaccination vertigo or ataxia (8/132; 6.0%) as detailed in Table 2. Less frequent etiologies included non-specific acute vertigo, Ramsay Hunt syndrome, stroke, cholesteatoma-related postsurgical vertigo, postoperative central nervous system disorders, tumors, encephalopathy, opsoclonus-myoclonus syndrome, and other isolated causes.

**Table 2:** Etiology of acute vestibular syndrome in the overall cohort.

Diagnosis	N	AVS %
Acute post-infectious cerebellitis	29	21.9
Vestibular neuritis	23	17.4
Post-traumatic vertigo	21	15.9
Sudden hearing loss	12	9.1
Labyrinthitis	11	8.3
Post-vaccination vertigo/ataxia	8	6.0
Acute vertigo, unspecified	6	4.6
CNS post-surgery	6	4.6
Ramsay Hunt syndrome	3	2.3
Stroke	3	2.3
CNS tumors	2	1.5
Cholesteatoma post-surgery	2	1.5
Other central causes	6	4.6
Total	132	100.0

Central causes, particularly post-infectious cerebellitis, accounted for the most frequent etiologies of AVS.

When AVS etiologies were classified into peripheral and central categories, central causes predominated overall, as shown in Table 3. Peripheral AVS accounted for 57 of 132 cases (43.2% [95%CI: 35.0–51.7]), whereas central AVS accounted for 75 of 132 cases (56.8% [95%CI: 48.3–65.0]).

**Table 3:** Distribution of acute vestibular syndrome according to peripheral or central etiologies.

Etiological group	N	% of AVS
Peripheral	57	43.2
Central causes	75	56.8
Total	132	100.0

Overall, central causes were more frequent than peripheral etiologies among patients with AVS.

Peripheral etiologies mainly included vestibular neuritis, sudden hearing loss, labyrinthitis, Ramsay Hunt syndrome, nonspecific acute vertigo, and cholesteatoma-related post-surgical vertigo. In contrast, central causes were predominantly represented by acute post-infectious cerebellitis, post-traumatic vertigo, post-vaccination vertigo or ataxia, postoperative central nervous system disorders, stroke, tumors, encephalopathy, opsoclonus-myoclonus syndromes, and other central or undetermined conditions.

### 3.2 Age-related differences in AVS etiology

The etiologic spectrum of AVS varied markedly according to age group, as detailed in Table 4. Among children aged 0–5 years, central causes clearly predominated, representing 28 of 38 cases (73.7%), while peripheral etiologies accounted for only 10 cases (26.3%). In the 6–11 years group, the distribution remained skewed toward central causes, with 32 of 53 cases (60.4%) classified as central causes and 21 (39.6%) as peripheral. In contrast, among adolescents aged 12–18 years, peripheral etiologies became predominant, accounting for 26 of 41 cases (63.4%), whereas central causes represented 15 cases (36.6%).

**Table 4:** Peripheral vs. central causes AVS according to age group.

Age group	Peripheral causes n (%)	Central causes n (%)	Total
0–5 years	10 (26.3)	28 (73.7)	38
6–11 years	21 (39.6)	32 (60.4)	53
12–18 years	26 (63.4)	15 (36.6)	41
Total	57 (43.2)	75 (56.8)	132

A clear age-dependent shift was observed, with central causes decreasing and peripheral etiologies increasing with age. Chi-square = 11.5; df = 2; *p* = 0.003.

A significant age-related shift was observed. The proportion of peripheral AVS increased progressively across age groups, from 26.3% [95%CI: 14.2–43.7] in children aged 0–5 years to 39.6% [95%CI: 27.4–53.2] in those aged 6–11 years and 63.4% [95%CI: 48.1–76.4] in adolescents aged 12–18 years (chi-square = 11.5; df = 2; *p* = 0.003). From a clinical perspective, these findings indicate that acute presentations in younger children are likely to be associated with central causes, whereas peripheral vestibular disorders become increasingly predominant during adolescence.

**Figure 3:**
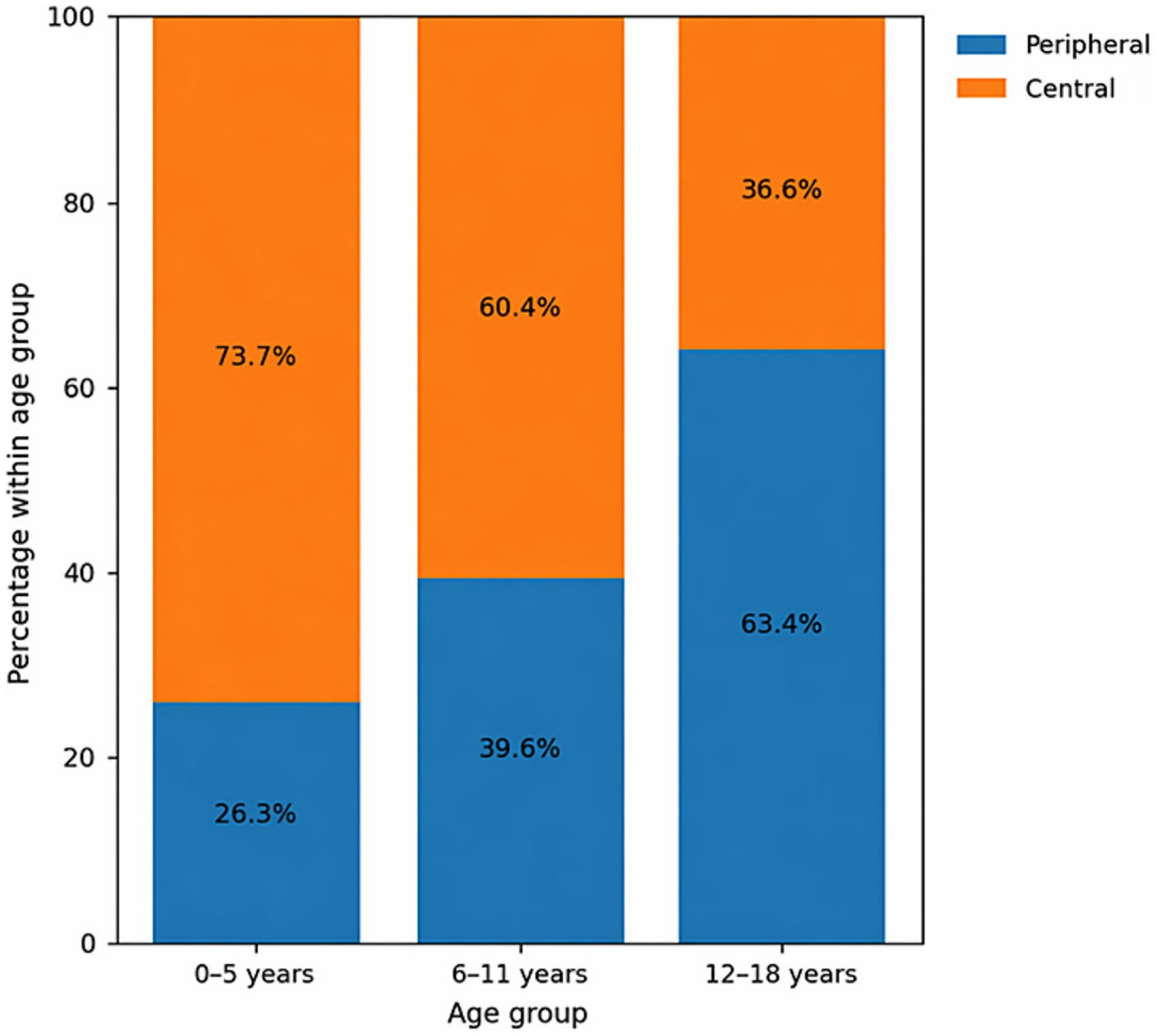
Distribution of peripheral and central causes of acute vestibular syndrome according to age group. The figure illustrates the progressive age-related shift inversion from a predominance of central etiologies in younger children to peripheral etiologies in adolescents.

As shown in Figure 3, the etiologic profile of AVS varied across age groups, with central causes predominating in younger children and a progressive increase in peripheral etiologies with advancing age.

### 3.3 Predominant diagnoses within each age group

In the 0–5 years age group, acute post-infectious cerebellitis was the leading diagnosis (19/38; 50.0%), followed by labyrinthitis (8/38; 21.1%) and post-traumatic vertigo (4/38; 10.5%). Among children aged 6–11 years, the etiological profile was more heterogeneous, with post-traumatic vertigo representing the most frequent diagnosis (12/53; 22.6%), followed by acute post-infectious cerebellitis (7/53; 13.2%), sudden hearing loss (7/53; 13.2%), vestibular neuritis (7/53; 13.2%), and stroke (1/53; 1.9%). In adolescents aged 12–18 years, vestibular neuritis was the predominant diagnosis (15/41; 36.6%), followed by post-traumatic vertigo (5/41; 12.2%), sudden hearing loss (4/41; 9.8%), acute post-infectious cerebellitis (3/41; 7.3%), and stroke (2/41; 4.9%; Table 5).

**Table 5:** Main diagnoses of acute vestibular syndrome according to age group.

Diagnosis	0–5 years (*n* = 38)	6–11 years (*n* = 53)	12–18 years (*n* = 41)
Acute post-infectious cerebellitis	19	7	3
Vestibular neuritis	1	7	15
Post-traumatic vertigo	4	12	5
Sudden hearing loss	1	7	4
Labyrinthitis	8	3	0
Post-vaccination vertigo/ataxia	1	5	2
CNS post-surgery	1	2	3
Ramsay Hunt syndrome	0	0	3
Stroke	0	1	2
Acute vertigo, unspecified	0	4	2
Cholesteatoma post-surgery	0	0	2
Other central causes	3	5	0

Diagnostic varied across age groups, with acute post-infectious cerebellitis predominating in younger children and vestibular neuritis in adolescents.

Taken together, these findings reinforce that AVS in pediatric patients should not be considered a homogeneous entity. In younger children, the differential diagnosis should primarily focus on centralcauses, particularly acute cerebellar syndromes and trauma-related disorders, whereas in adolescents, peripheral vestibular disorders emerge as the predominant diagnostic consideration.

In addition, eight patients presented with acute vestibular symptoms temporally associated with vaccination, occurring approximately 2 to 3 weeks after vaccine administration. The vaccines involved were Sinopharm (4/8), the measles–mumps–rubella vaccine (MMR; 3/8), and human papillomavirus vaccine (1/8). Alternative potential causes of the presenting symptoms were considered and excluded based on the available clinical evaluation.

Given the small number of cases, these observations are presented descriptively and should be interpreted with caution.

## 4 Discussion

Our results are in line with previous reports highlighting the heterogeneity of vertigo in the pediatric population, although most published series have focused on overall vestibular disorders rather than specifically on AVS. Studies such as those by Yılmaz et al. (6) and Raucci et al. (7) describe vestibular migraine, benign paroxysmal vertigo of childhood, and functional disorders as the most frequent diagnoses; however, these conditions correspond to episodic vestibular syndromes according to the Bárány classification. In contrast, by specifically restricting our analysis to AVS, we identified a distinct etiological profile characterized by a predominance of acute cerebellitis and post-traumatic disorders, particularly among younger patients.

The predominance of central causes in early childhood may be explained by several factors. Acute post-infectious cerebellitis, the leading diagnosis among younger children in our series, is a well-recognized condition associated with viral infections and characterized by the acute onset of ataxia and imbalance. It is the most common cause of ataxia in early childhood. It typically occurs between the first and third week following a febrile infectious illness. The diagnosis is clinical and remains a diagnosis of exclusion.

In children younger than 18 months, labyrinthitis may arise as a complication of acute otitis media (8, 9).This condition often goes unrecognized in non-ambulatory patients, but it should nevertheless be considered in the differential diagnosis. In most cases, symptoms are resolved with appropriate antibiotic treatment; however, because labyrinthitis may result in hearing loss, audiological follow-up is recommended when this diagnosis is suspected.

In this large pediatric cohort, we identified a clear age-dependent shift in the etiological profile of AVS. The main finding was the predominance of central causes in younger children, whereas peripheral vestibular disorders became progressively more frequent in older children and adolescents. This pattern was statistically significant and clinically consistent across all age groups.

In adult populations, AVS of central origin is most commonly associated with posterior fossa stroke, particularly cerebellar or brainstem infarction, which constitutes the main diagnostic concern in emergency settings. In contrast, only three confirmed cases of ischemic or hemorrhagic stroke were identified as causes of AVS in our pediatric cohort.

The predominant central etiology in our cohort was acute post-infectious cerebellitis, accounting for 21.9% of all AVS cases and for 50% of cases in the youngest age group (0–5 years). This finding is consistent with the low incidence of stroke in the pediatric population and highlights that the diagnostic framework for central AVS in children differs substantially from that of adults and cannot be directly extrapolated from adult neurology. Accordingly, clinicians evaluating children with acute vertigo and central signs should primarily consider infectious and parainfectious cerebellar disorders, while remaining alert to the rare but clinically significant possibility of posterior circulation stroke (10, 11).

Balance disturbances following trauma typically present acutely. A history of trauma is usually easily identifiable, and symptoms often improve spontaneously or, in more symptomatic cases, with the use of symptomatic medication. In our cohort, the prevalence of post-traumatic vestibular disorders was similar across all age groups, without statistically significant differences. Most cases were associated with mild head trauma, for which the terms labyrinthine concussion or post-traumatic labyrinthine dysfunction would be more appropriate. These conditions are considered to result from a temporary disruption of the cochleovestibular receptors (12).Only one patient in our series sustained severe trauma, consisting of a temporal bone fracture and subsequent hearing loss.

Vestibular neuritis is an acute inflammatory disorder that primarily affects the superior vestibular nerve, although the inferior branch or both branches may be involved unilaterally. It often has a viral origin and typically presents with an acute onset of prominent vestibular symptoms (13). This condition predominantly affects young adults and children older than 10 years. Balzanelli et al. (14)evaluated a series of 472 patients over a 9-year period and identified neuritis/labyrinthitis (AVS) as the most frequent cause of vertigo in pediatric patients (26.1%), surpassing vestibular migraine (21.2%) and benign paroxysmal positional vertigo (BPPV; 10.2%). BPVC accounted for only 8.5% of cases. We believe that these differences in etiological distribution may be explained by the older age of their cohort (11 ± 3), corresponding to patients in the older age groups according to our classification.

In accordance with the Bárány Society classification, we specifically emphasized diagnoses associated with acute vestibular presentations in children. Other studies have defined acute vertigo more broadly as any vertiginous symptom leading to presentation to the emergency department (15–17). Nevertheless, in the present study, diagnoses such as BPPV, BPVC, and migraine, even when presenting as a first episode, were classified as episodic vestibular syndromes rather than acute vestibular disorders. Consequently, these entities were not included in the AVS subgroup analysis.

This study has several limitations. It is a retrospective study, and cases were derived from a single otorhinolaryngology department within a tertiary referral hospital, which may limit the generalizability of the findings and potentially overrepresent more complex or severe conditions compared with the general pediatric population. Furthermore, because formal vestibular testing could not always be performed in the youngest children, diagnostic certainty may have differed slightly across age groups.

Despite these limitations, this study has several important strengths, including its large sample size and the use of a standardized vestibular classification system combined with age-stratified analysis, which allowed a focused characterization of AVS in the pediatric population. Future prospective studies are needed to validate these findings and to further refine age-specific diagnostic algorithms.

## Data Availability

The raw data supporting the conclusions of this article will be made available by the authors, without undue reservation.
